# Comparative Study of the Corrosive Behaviors of Rust Layers on Bronze Ware in Different Corrosive Environments

**DOI:** 10.3390/ma18061359

**Published:** 2025-03-19

**Authors:** Bingbing Li, Qixing Xia, Wenqiang Dong

**Affiliations:** 1 Cultural Heritage Research Institute, Northwestern Polytechnical University, Xi’an 710072, China; 2 Key Laboratory of Archaeological Exploration and Cultural Heritage Conservation Technology (Northwestern Polytechnical University), Ministry of Education, Xi’an 710129, China

**Keywords:** bronze ware, rust layers, electrochemical analysis, corrosive environment

## Abstract

It is of great significance to clarify the corrosion mechanism of rust layers on bronze ware for appropriate conservation measures. In this study, the corrosion behavior of Cu-Sn bronze alloys in a 3.5 wt.% NaCl solution and a simulated archaeological soil solution was studied and compared using electrochemical measurements, microscopic observations, X-ray diffraction (XRD), and X-ray photoelectron spectroscopy (XPS). The results showed that the presence of Cl^−^ was the key factor leading to the formation of harmful rust such as Cu_2_(OH)Cl_3_. In the NaCl solution, the rapid accumulation of Cl-containing corrosion products provided a certain degree of protection to Cu-Sn alloys, but the products easily fell off, thus increasing the continuous corrosion reactions again. This resulted in a significant increase in the corrosion rate of the alloy (*i*_corr_ from 4.845 μA·cm^−2^ to 27.21 μA·cm^−2^) and a decrease in polarization resistance (*R*_p_ from 5.17 kΩ·cm^2^ to 3.27 kΩ·cm^2^). In contrast, the corrosion reactions of the Cu-Sn alloy were dominated by complex ions other than Cl^−^ in archaeological soil environments, and the corrosion products tended to form stable and dense rust layers (*i*_corr_ was always lower than 1.6 μA·cm^−2^, and *R*_p_ was maintained above 24 kΩ·cm^2^), which improved corrosion resistance by two orders of magnitude compared to the unstable rust layer that formed in NaCl solution. In addition, Cl-containing corrosion products boosted the wettability of rust layers, thereby facilitating penetration of corrosive media that strengthened corrosion reactions. This study deepens our understanding of the degradation mechanisms of bronze artifacts and provides a scientific basis for developing bronze conservation strategies.

## 1. Introduction

Bronze, the first metal mastered by humans, possesses superior mechanical properties and durability compared to materials such as pottery and stone, significantly advancing human technological capabilities [1]. With the widespread adoption of bronze in various ancient civilizations, including Egypt, Babylon, and China, human society entered the “Bronze Age” [2]. The chemical composition of bronze primarily consists of a copper-tin alloy, often containing trace amounts of lead and other metallic elements derived from ores. When initially cast, the bronze alloy has a golden yellow color and metallic sheen. In comparison, most bronze ware is exposed to the erosive effects of moisture, air, and corrosive ions when buried in soil, leading to the formation of corrosion products on the bronze surface, commonly referred to as patina [3]. Patina can appear in various colors, including green, blue, brown, and red, depending on the predominant corrosion compounds present [4,5]. Not only does patina enhance the artistic and aesthetic value of bronze, but it also acts as a protective layer, shielding the underlying material from further external corrosion.

Patina represents a dynamic corrosion equilibrium between bronze ware and its surrounding environments [6,7,8]. However, when powdery rust forms within the patina, this equilibrium is easily disrupted, leading to reactivation of the corrosion process in the bronze. Chloride corrosion products, such as Cu_2_(OH)Cl_3_ and CuCl, are the primary components of the powdery rust on bronze [9,10,11,12]. These products can further accelerate corrosion, causing the rust layer to become loose and swollen, and ultimately resulting in the bronze crumbling into a powder [11,13]. Previous studies have extensively investigated the corrosion behavior of Cu-Sn bronze in different environments, particularly the stability of the corrosion layer and the influence of chloride ions on the formation of powdery rust [12,14,15,16,17]. Song Z [18] studied the corrosion behavior of Cu-20 wt.% Sn bronze alloy in NaCl solutions of varying concentrations and found that the α phase was highly susceptible to corrosion in NaCl solution, whereas the δ phase, with its higher Sn content, exhibited excellent corrosion resistance. Liang Z [19] examined the corrosion behavior of synthetic Cu-Sn bronze alloys with six different Sn contents in a soil simulation solution. The study found that, when the corrosion products mainly consisted of metal-insoluble inert compounds and bound water, the corrosion layer provided an effective barrier to the penetration of corrosive ions (SO_4_^2^⁻ and Cl⁻) into the alloy matrix. Hu Y [20], by analyzing the microstructure of copper artifacts and the burial microenvironment, concluded that chloride ions play a crucial role in the formation and transformation of bronze corrosion products by increasing the solubility of the malachite layer and enhancing the conductivity of the substrate. These studies collectively underscore the significance of environmental factors, such as chloride ion concentration and the presence of multiple ions in the medium, in controlling the corrosion behavior of Cu-Sn alloys and the formation of powdery rust.

Current research on the treatment of powdery rust on bronze primarily focuses on chemical corrosion inhibitors and protective coatings [21,22]. These methods work by forming a protective film on the bronze surface, which blocks the continuous supply of environmental factors such as Cl^−^, H_2_O, and O_2_, all of which drive the corrosion process. Kosec et al. developed a corrosion inhibitor, tolyl methyl imidazole (TMI), to prevent bronze corrosion under indoor storage conditions [23]. Wu et al. proposed a novel strategy for protecting bronze cultural relics based on photo-induced passivation [24,25] that uses ultraviolet light to form a dense SnO_2_ passivation layer within the rust on the bronze surface, thereby significantly reducing corrosion reactions. However, the effectiveness of these protective measures largely depends on the characteristics of the rust layer [9,26,27], making it crucial to understand the formation mechanisms of various corrosion products, particularly powdery rust, in buried environments over time.

In this study, we prepared a simulated archaeological soil solution based on the soluble ion composition of archaeological soil around bronze ware unearthed in Shaanxi, and subsequently investigated the corrosion behavior of the Cu-Sn alloy in this simulated archaeological soil solution medium. Given that Cl- is a key factor in the corrosion of bronze and the formation of powdery rust [16,20,28], we also used NaCl solution as a control to systematically analyze the formation mechanisms of corrosion products such as Cu_2_(OH)Cl_3_. Furthermore, we evaluated the influence of roughness and wettability on the corrosion reactions and compared progression of the corrosion process on the surface of bronze ware in the archaeological soil medium and NaCl solutions through electrochemical tests. This work will not only provide theoretical guidance for understanding the corrosion behavior of bronze ware in soil, but will also have positive significance for the effective treatment of powdery rust on bronze ware.

## 2. Experimental Work

### 2.1. Materials

The materials tested in this study are Cu-Sn alloys, and the elemental compositions are presented in Table S1. Cu-Sn alloys were sectioned into 10 mm × 10 mm × 1 mm samples for corrosion treatment and electrochemical testing. Prior to the experiment, the samples were mechanically polished using SiC papers (P800, P1000, P2000, P2500, and P5000 in sequence), with deionized water serving as the polishing medium. After polishing, the samples were ultrasonically cleaned in an ethanol bath, followed by washing with distilled water and air-drying at room temperature.

### 2.2. Simulated Corrosion Treatments

To simulate the burial environment of cultural relics, a simulated archaeological soil solution was prepared based on the soluble ion composition of buried soil around bronze ware unearthed in Shaanxi, as shown in Table S2. The control experiment used 3.5 wt.% NaCl solution as the corrosion medium. At 20 °C, the NaCl solution has a pH value of 7.1 and a conductivity of 4.8 S/m, while the simulated archaeological soil solution had a pH value of 7.8 and a conductivity of 4.6 S/m. The Cu-Sn alloys were immersed in the simulated archaeological soil solution or NaCl solution for 0.5 h, 2 h, 6 h, 12 h, or 24 h, as shown in Table 1.

### 2.3. Electrochemical Corrosion Tests

Electrochemical experiments were performed with a CHI604F workstation (Shanghai Chenhua Instruments Co., Ltd., Shanghai, China) in a three-electrode cell. Specifically, the sample was the working electrode, platinum was the counter electrode, and a saturated calomel electrode (SCE) was the reference electrode. The active surface area of the samples was 1 cm^2^. The volume of electrolyte solution was 350 mL. The electrochemical impedance spectra (EIS) of the Cu-Sn alloy were recorded over the frequency range of 100 kHz to 0.1 Hz, with a 10 mV AC signal applied at the corrosion potential (*E_corr_*). The number of frequency points per decade was 10. The obtained EIS data were fitted to a 2RC equivalent circuit model using ZSimpWin3.3 software. The Tafel curves were obtained at ±0.25 V vs. *E_corr_* at a scan rate of 1 mV/s.

### 2.4. Characterization Methods

#### 2.4.1. Contact Angle Measurement and Roughness Tester

Contact angle measurements were conducted using an LSA100 optical contact angle goniometer (LAUDA Scientific GmbH, Königshofen, Germany) at an ambient temperature range of 22~25 °C and a relative humidity of 20~40%. Distilled water was used as the test liquid. A 10 μL droplet was formed at the end of the syringe and precisely deposited onto the sample surface. The syringe was then lifted while simultaneously capturing video footage. The contact angle was calculated using the TrueDrop model in the Surface Meter™ software version 1.2.2.18 program. Each sample was measured in triplicate, and the average value was taken. Surface roughness measurements were performed on the sample surface using a TR200 handheld surface roughness tester (Beijing Time Sun Science&Trade Co., Ltd., Beijing, China), with a precision of 0.001 μm and a driving stroke length of 1.3 mm. For each sample, three readings were recorded, and the average value was calculated.

#### 2.4.2. Morphology Observations

The surface morphology of the corroded samples was observed using an upright metallographic microscope (SOPTOP R×50M, Ningbo Sunny Instruments Co., Ltd., Ningbo, China) and a scanning electron microscope (SEM, ZEISS Sigma 300, Carl Zeiss AG, Oberkochen, Germany) capable of X-ray energy dispersive spectroscopy (EDS).

#### 2.4.3. Composition Characterizations

X-ray diffraction (XRD, Bruker D8 Advance, Bruker Corporation, Billerica, MA, USA) was employed to analyze the composition of the corrosion products. The diffraction intensity of Cu Kα radiation (λ = 0.1542 nm) at 40 kV and 40 mA was recorded over the 2θ range of 5° to 90°, with a scanning speed of 5°/min. The test results were analyzed and fitted using JADE 6.5 software. The composition elements on the corroded surfaces of the samples were studied by X-ray photoelectron spectroscopy (XPS, Thermo Scientific K-Alpha, Thermo Fisher Scientific, Waltham, MA, USA). All XPS peaks were referenced to the standard carbon C 1s binding energy (284.8 eV), and the data were fitted using XPSPEAK 4.1 software, with background subtraction conducted using the Tougaard method.

## 3. Results and Discussion

### 3.1. Structural Characterization

#### 3.1.1. Metallographic Analysis

**Figure 1:**
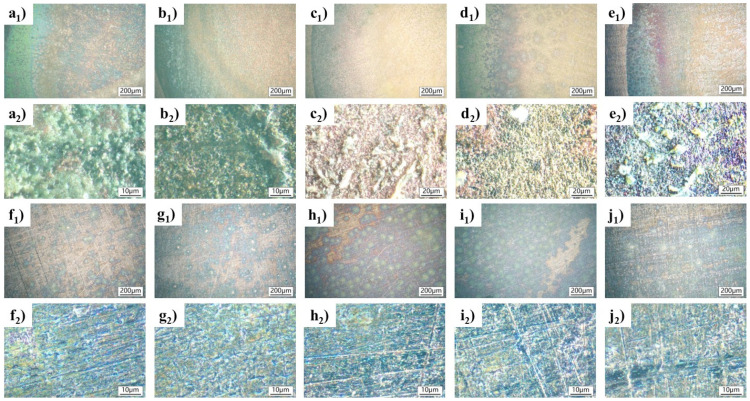
Surface morphology of Cu-Sn alloys after immersing in NaCl solution and simulated archaeological-soil solution: (**a_1_**,**a_2_**) Cl-0.5, (**b_1_**,**b_2_**) Cl-2, (**c_1_**,**c_2_**) Cl-6, (**d_1_**,**d_2_**) Cl-12, (**e_1_**,**e_2_**) Cl-24; (**f_1_**,**f_2_**) SS-0.5, (**g_1_**,**g_2_**) SS-2, (**h_1_**,**h_2_**) SS-6, (**i_1_**,**i_2_**) SS-12, (**j_1_**,**j_2_**) SS-24.

Figure 1 shows metallographic photos of the alloys after immersion in the NaCl and simulated archeological soil solutions. A 12V 50W halogen lamp was used as the light source. The surface of the untreated Cu-Sn alloy (Figure S1) displayed a relatively uniform alloy microstructure, with no visible corrosion products or localized damage. From Figure 1(a_1_–j_1_), it can be seen that the corrosion morphologies of the Cu-Sn alloys in the 3.5% NaCl solution became more and more serious over time. As for Cl-0.5, slight corrosion signs began to appear on the surface, with a few uneven areas, possibly indicating the early stages of pitting corrosion. After 2 h of corrosion, the corroded areas expanded, and surface oxidation intensified, suggesting further development of pitting corrosion (Cl-2). After 6 h of corrosion, the corrosion products covered most of the metal surface, and the corrosion products gradually formed a flaky structure, resulting in the more uniform and rougher corroded surface. When the treatment time was longer than 12 h, the corrosion products completely covered the surface, and deeper localized corrosion damage appeared, likely due to corrosion pits merging.

The corrosion morphologies of the Cu-Sn alloys in archeological soil environments were characterized over time (Figure 1(a_2_–j_2_)). Compared to the 3.5 wt.% NaCl solution, the simulated archeological soil contained very few of Cl^−^ ions, and the corrosion states of the Cu-Sn alloys were relatively weaker (SS-0.5 to SS-24). Specifically, very light, uneven corrosion signs appeared on SS-0.5, and the corrosion developed very slowly over time. When the treatment time was increased to 6 h, the corrosion products became complex, with some regions developing a continuous rust layer. Cl-12 and Cl-24 exhibited similar corrosion states, with the generation of a thin rust layer, indicating that the corrosion had reached a relatively stable stage. Despite small holes possibly occurring in some areas, the corrosion products that formed in the simulated archeological soil solution were denser and thinner than those that formed in the NaCl solution, and the corrosion processes varied much more, owing to the presence of several types of ions.

#### 3.1.2. SEM and EDS Analysis

**Figure 2:**
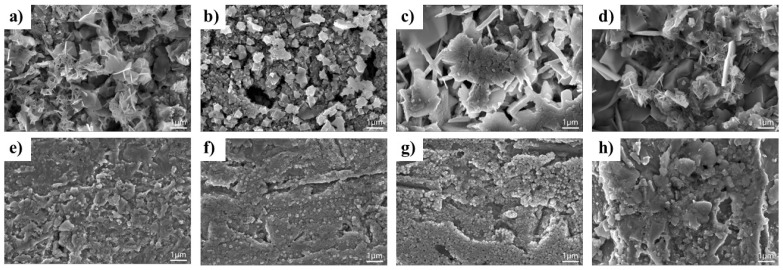
SEM micrographs of Cu-Sn alloy surfaces after immersion in a NaCl solution or a simulated archaeological soil solution: (**a**) Cl-0.5, (**b**) Cl-2, (**c**) Cl-6, (**d**) Cl-12; (**e**) SS-0.5, (**f**) SS-2, (**g**) SS-6, (**h**) SS-12.

SEM was employed to studied the corrosion morphologies of Cu-Sn alloys in different environments at high magnification, as show in Figure 2. In the NaCl solution, the corrosion products of Cl-0.5 exhibited a flaky morphology, indicative of the initial formation of oxidation and chloride compounds, such as Cu_2_O or CuCl, on the alloy surface. Localized agglomeration of corrosion products was observed on the surface of Cl-2 and Cl-6, forming clusters and leaving behind regions with porous structures, suggesting the progression of pitting corrosion with localized corrosion sites. When the immersion time was increased to 12 h, the rust layer on Cl-24 developed a lumpy and highly porous structure, reflecting continuous deposition and detachment of corrosion products due to Cl^−^ penetration and localized anodic dissolution. Generally, the corrosion processes of Cu-Sn alloys in the NaCl solution were dominated by Cl^−^ inducing breakdown of the passive layer, as well as the cyclic formation/dissolution of corrosion products. The loose and porous nature of the rust layer contributed to further degradation of the alloy, underscoring its susceptibility to long-term exposure to chloride-rich environments.

The corrosion behavior of Cu-Sn alloys in an archeological soil environments exhibited a more stable trend (Figure 2e–h). During the initial immersion stage (0.5 h), corrosion on the alloy surface was relatively mild, with only a small amount of discontinuous corrosion products observed, and the substrate surface remained relatively intact. As corrosion progressed to 2 h, the corrosion products gradually increased, forming a particulate structure. After 6 and 12 h, the corrosion product layer continued to accumulate, forming a relatively smooth and uniform covering. The surface exhibited dense packing of nanoscale particles, indicating that the corrosion product layer that formed in the simulated soil solution was more stable, which helped slow further penetration of corrosive media.

**Figure 3:**
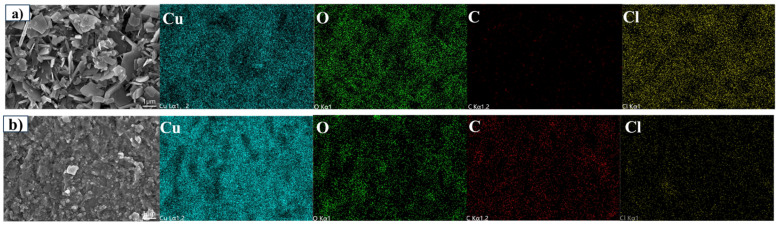
SEM micrographs and EDS mapping analysis of Cu-Sn alloys surface after immersion treatment for 24 h: (**a**) Cl-24, (**b**) SS-24.

Figure 3 displays the SEM morphologies and EDS mapping results for Cl-24 and SS-24. Significant corrosion occurred on the surface of Cl-24, forming a loose and porous corrosion rust layer. The corrosion products primarily consisted of aggregated structures with varying particle sizes, corresponding to inhomogeneous corrosion behaviors in NaCl solutions. Moreover, the porosity of the rust layer suggests that it could not form an effective protective barrier, allowing Cl^−^ to penetrate continuously and accelerate the corrosion reaction. EDS mapping showed widespread Cl^−^ in the corrosion products, demonstrating the formation of copper chloride–based corrosion compounds in Cl^−^-rich environments. Table S3 shows the effects of treatment time on EDS element composition. The Cl^−^ content of the rust layers initially increased and then decreased, which might be due to the continuous accumulation of Cl^−^-containing corrosion products in the early stage, followed by a certain degree of detachment in the later stage. Combining with SEM analysis, it can be inferred that the rust layers failed to form a dense structure in NaCl solution, leading to long-term active corrosion.

In contrast, SS-24 exhibited a relatively smooth particulate corrosion product layer (Figure 3b), and the distribution of the surface products was relatively uniform, with no noticeable pores or cracks. This suggests that the corrosion product layer that formed in the simulated archaeological soil solution has a certain degree of integrity and protective coverage, which helps to prevent further penetration of the solution. Notably, the distribution of Cl^−^ was sparse, with low intensity, indicating that Cl^−^ ions played a lesser role in corrosion. By comparing the corrosion behavior of the alloy in two different corrosive environments, it could be determined that a high concentration of Cl^−^ in surrounding environments resulted in the formation of numerous flaky corrosion products, which could facilitate penetration of Cl^−^ into the rust layer and promote the transition from pitting corrosion to general corrosion. When the concentration of other ions in the environment was higher, and Cl^−^ was relatively scarce, the rust layer that formed on the alloy exhibited a dense and stable structure, which played a positive role in inhibiting further corrosion.

#### 3.1.3. Contact Angle and Roughness Analysis

**Figure 4:**
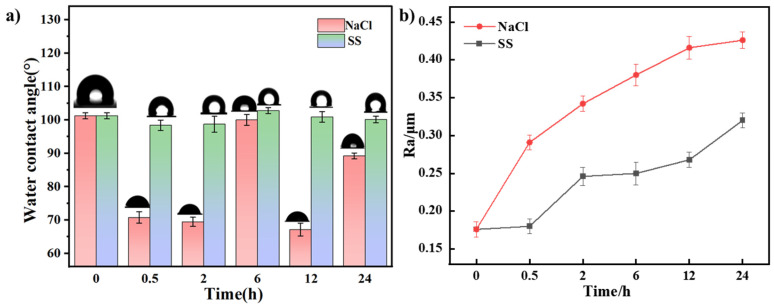
Variations in contact angle (**a**) and surface roughness (**b**) of Cu-Sn alloys after immersion in a 3.5% NaCl solution or a simulated archaeological soil solution (SS).

Figure 4 shows the surface wettability and roughness of Cu-Sn alloys over time in 3.5 wt.% NaCl solution and simulated archeological soil solution (SS). As shown in Figure 4 a, with the extension of corrosion time, the contact angle on the alloy surface in both corrosion mediums exhibited an overall decreasing trend. In the NaCl solution, during the initial corrosion stages (0.5 h and 2 h), the contact angle decreased from 101.2° to 69.45°, signifying a significant increase in the wettability of the alloy surface, which might facilitate penetration of the corrosive medium into the alloy substrate. The relatively strong wettability mainly was attributed to the formation of loose and porous chloride-rich products on the surface. In the archeological soil environment, the decrease in contact angle was slower and smaller, which might be due to the denser corrosion product layer.

The change in surface roughness (*R_a_*) (Figure 4b) further confirmed this trend. In the NaCl solution, the *R_a_* value rapidly increased to about 0.426 μm within 24 h, indicating that the porous structure of the corrosion product layer on the surface significantly increased the corrosion reaction area. In the simulated archeological soil solution, the increase in *R_a_* was relatively slow, suggesting that the denser corrosion product layer effectively limited the changes in surface roughness. Therefore, the decrease in contact angle and the increase in surface roughness suggest that there are significant differences in the formation mechanisms of corrosion product layers in different corrosion media: the rust layer formed in the NaCl solution was loose and porous, promoting enhanced wettability and a rapid increase in surface roughness, while the rust layer formed in the simulated archaeological soil solution was denser, effectively slowing down these changes in wettability and roughness.

### 3.2. Chemical Compositions

#### 3.2.1. XRD Analysis

**Figure 5:**
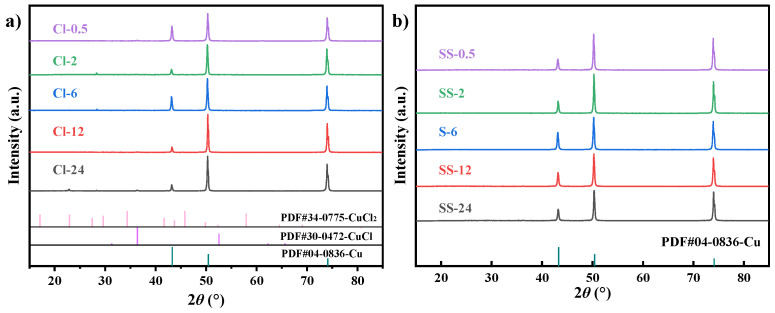
XRD analysis results of Cu-Sn alloys after immersion in (**a**) a 3.5% NaCl solution or (**b**) a simulated archaeological soil solution.

Figure 5 shows the X-ray diffraction (XRD) spectra of the Cu-Sn alloys after different corrosion times in two corrosion media. In the NaCl solution, the XRD spectra indicate that the corrosion products were relatively few, with diffraction peaks of CuCl and CuCl_2_ primarily appearing at 24 h. As corrosion time increased, the diffraction peaks of the Cu-based alloy gradually weakened, suggesting that corrosion of the substrate intensified. In contrast, aside from the diffraction peak of the Cu substrate, no distinct corrosion product diffraction peaks were detected in SS-0.5 to SS-24, which suggested that the amount of corrosion products formed during the corrosion process was relatively small, with no significant crystalline corrosion products formed. The difference in corrosion behavior between the two media highlights the dominant role of Cl^−^ concentration in the corrosion mechanism. In the NaCl solution, the high concentration of Cl^−^ accelerated pitting corrosion of the alloy substrate and dissolution/redeposition of corrosion products, leading to a loose and discontinuous rust layer. In contrast, the low concentration of Cl^−^ and the competitive effects of various anions in the simulated archaeological soil solution slowed the corrosion rate, while also suppressing the formation of crystalline corrosion products, thereby forming a dense, uniform protective rust layer on the surface.

#### 3.2.2. XPS Analysis

**Figure 6:**
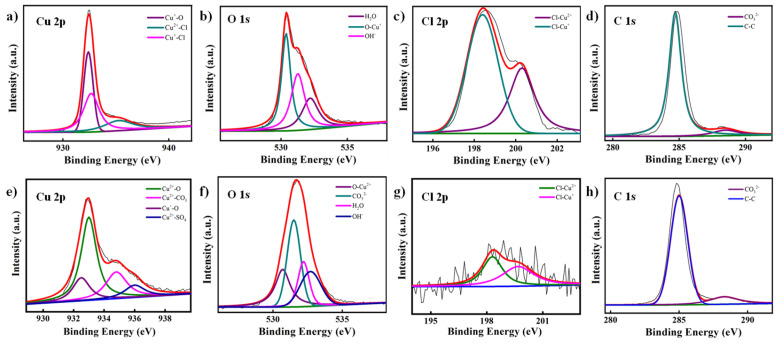
High-resolution XPS results from Cu 2p, O 1s, Cl 2p, and C 1s on Cu-Sn alloys after immersion in (**a**–**d**) a 3.5% NaCl solution or (**e**–**h**) a simulated archaeological soil solution.

In order to further determine the composition of the rust layers, XPS was used to study the element states of Cl-24 and SS-24, as shown in Figure 6. As for Cl-24, there were three main peaks at 932.4 eV, 935.25 eV, and 932.67 eV observed for Cu 2p XPS, corresponding to Cu⁺-O, Cu^2^⁺-Cl, and Cu⁺-Cl, respectively [29,30]. Additionally, three peaks existed in the O 1s XPS, at 532.8 eV, 530.38 eV, and 531.27 eV, that were assigned to water (H_2_O), copper(I) oxide (Cu_2_O), and hydroxide (OH^−^) [31,32]. The Cl 2p XPS displayed two peaks at 200.3 eV and 198.4 eV, corresponding to Cl-Cu^2+^ and Cl-Cu⁺ [33,34], indicating that the reaction of Cl^−^ with copper likely produced copper chlorides (CuCl, CuCl_2_, and Cu_2_(OH)_3_Cl), similar to the XRD results. C 1s XPS revealed two peaks at 288.5 eV and 284.7 eV, attributed to CO_3_^2−^ and contaminant carbon, respectively [35,36]. Cl-24 absorbed a small amount of CO_2_ from the air, then CO_3_^2−^ was formed. In addition, corrosion in the NaCl solution not only promoted the formation of copper chlorides but also generated hydrated oxides, which further influenced the chemical environment of the alloy surface and increased wettability.

As for SS-24, the Cu 2p XPS exhibited four main peaks at 933 eV, 934.8 eV, 932.5 eV, and 936.0 eV, corresponding to Cu^2+^-O, Cu^2+^-CO_3_, Cu^+^-O, and Cu^2+^-SO_4_, respectively [37,38,39]. Compared to Cl-24, the Cu^2+^-O and Cu^+^-O of SS-24 were more distinct, and the presence of copper sulfate compound was observed, resulting in an increase in the passivation performance of the rust layer on SS-24. O1s showed four peaks (530.7 eV, 531.5 eV, 532.8 eV, and 532.7 eV), corresponding to CuO, CO_3_^2−^, H_2_O, and OH^−^, respectively [31,33,40,41]. Additionally, the Cl 2p spectrum revealed two peaks (198.4 eV and 199.68 eV), corresponding to Cl-Cu⁺ and Cl-Cu^2^⁺, respectively [34,42], with lower peak intensities, indicating a lower concentration of Cl^−^ in the archeological soil environment. The C 1s spectrum displayed two peaks at 288.5 eV and 284.7 eV, attributed to CO_3_^2−^ and contaminant carbon, respectively [35,36], with the CO_3_^2^⁻ peak being more pronounced, further confirming the significant formation of carbonate on SS-24. From the above analysis, it was concluded that copper chloride (CuCl_2_) and copper(I) oxide (Cu_2_O) were the predominant products in the NaCl environment (Cl-24), and the effect of Cl^−^ ions on the corrosion process were more pronounced. In contrast, the proportion of copper(II) oxide (CuO) was higher, and the corrosion product layer was denser, in the archeological soil environment (SS-24). Owing to the lesser effect of Cl^−^ during the immersion process, the rust layer formed in the archeological soil environment exhibited a certain degree of corrosion resistance.

### 3.3. Electrochemical Corrosion Behaviors

#### 3.3.1. Open Circuit Potential (OCP) Curves

**Figure 7:**
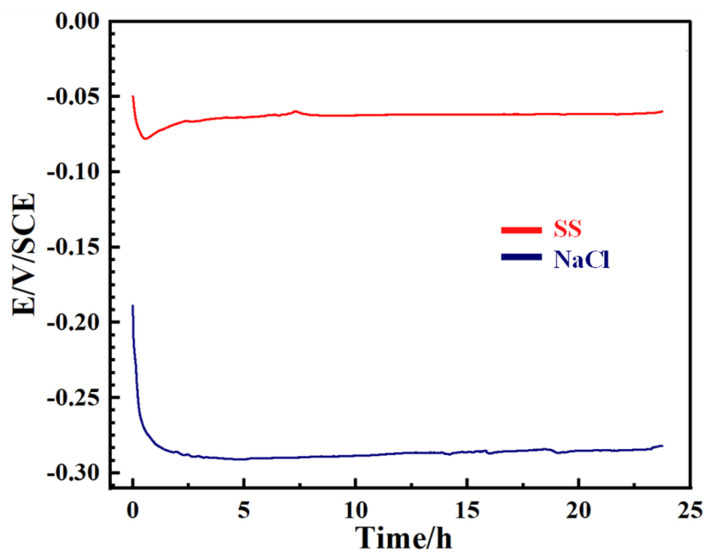
OCP curves of the Cu-Sn alloy over time in a 3.5% NaCl solution or a simulated archaeological soil solution (SS).

Figure 7 illustrates the OCP curves of the Cu-Sn alloy during immersion in a 3.5% NaCl solution or a simulated archaeological soil solution. In the NaCl solution, the OCP rapidly decreased during the initial stage (0~0.5 h), indicating that the Cu-Sn alloy surface experienced intense pitting corrosion. Over the next 0.5–24 h, the OCP gradually stabilized. This rapid decrease followed by stabilization suggests that the high concentration of Cl^−^ had a significant impact during the early stage of corrosion, leading to breakdown of the passive film and promoting substrate dissolution. However, as the immersion time increased, the sample surface became completely covered by the passive film, reaching a stable state. In comparison, in the simulated archaeological soil solution, OCP exhibited a slower decline. This gradual potential change indicates a more moderate corrosion process, possibly due to the formation of a more stable passivation layer. However, slight fluctuations in the OCP suggest that the growth and localized dissolution of the passivation film were still ongoing.

#### 3.3.2. Tafel Polarization Curves

**Figure 8:**
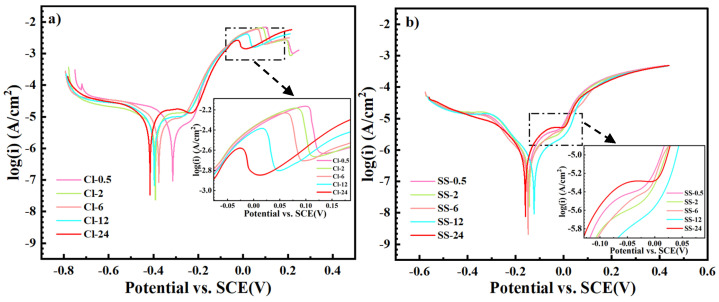
Tafel polarization curves for the Cu-Sn alloys in (**a**) a 3.5% NaCl solution or (**b**) a simulated archaeological soil solution.

Figure 8 shows the Tafel polarization curves of the Cu-Sn alloys in a 3.5% NaCl solution or a simulated archaeological soil solution. In the NaCl solution, as the corrosion time increased, the *E*_corr_ of the alloy gradually shifted more negatively, indicating an enhancement of corrosion activity. In the anodic region of the polarization curve, a significant increase in current density was observed, indicating that the alloy surface underwent intense anodic dissolution, with chloride ions contributing significantly to this process. In the simulated archeological soil solution, the polarization behavior of the Cu-Sn alloys exhibited different characteristics than those seen in the NaCl solution. The *E*_corr_ remained relatively stable, initially around −0.073 V/SCE, and only slightly decreased to −0.060 V/SCE after 24 h. The current density in the polarization curve was relatively low, suggesting that the corrosion process was suppressed, and the formation of the corrosion product layer effectively reduced further dissolution of the metal substrate. The great difference in corrosion current density could prove the corrosion inhibition effect of the simulated archaeological soil solution on Cu-Sn alloys.

Based on the polarization parameter data listed in Table 2, we further analyzed the corrosion behavior of Cu-Sn alloys in different corrosion mediums. The Stern–Geary equation was used for quantitative estimation of the corrosion current density (*i*_corr_) and polarization resistance (*R*_f_) [43,44]:

$$i_{corr}=\frac{1}{2.3Rp}\left(\frac{\beta_{a}|\beta_{c}|}{\beta_{a}+|\beta_{c}|}\right)=\frac{B}{R_{p}}$$

In the NaCl solution, the *i*_corr_ value gradually increased from 4.845 μA·cm^−2^ (Cl-0.5) to 27.21 μA·cm^−2^ at (Cl-24), indicating a sharp increase in the corrosion rate. In contrast, the *i*_corr_ value in the simulated archeological soil solution showed only a slight change, varying from 1.441 μA·cm^−2^ (SS-0.5) to 1.588 μA·cm^−2^ at (SS-24), suggesting that the simulated archaeological soil solution had a more effective inhibitive effect on corrosion. In the simulated archeological soil solution, the minimum corrosion current density (*i*_corr_) value occurred at SS-6 (0.796 μA·cm^−2^). This may be due to the formation of a dense and stable rust layer (possibly containing Cu_2_(OH)_2_CO_3_ and CuO) at 6 h (SS-6), which reduced exposure of the alloy surface to the corrosive medium. The *Rₚ* value in NaCl solution decreased from 5.17 kΩ·cm^2^ to 3.27 kΩ·cm^2^, indicating the loose structure of the corrosion product layer. In the simulated archaeological soil solution, the *Rₚ* value remained relatively high, signifying that the corrosion product layer was denser and effectively inhibited progression of corrosion. Thus, the corrosion rate of Cu-Sn alloys in the NaCl solution was 30–50 times higher than that in the simulated archaeological soil solution. In other words, the rust layer that formed in the simulated soil solution exhibited a corrosion resistance that was two orders of magnitude higher than that of the rust layer that formed in the NaCl solution.

#### 3.3.3. EIS Curves

**Figure 9:**
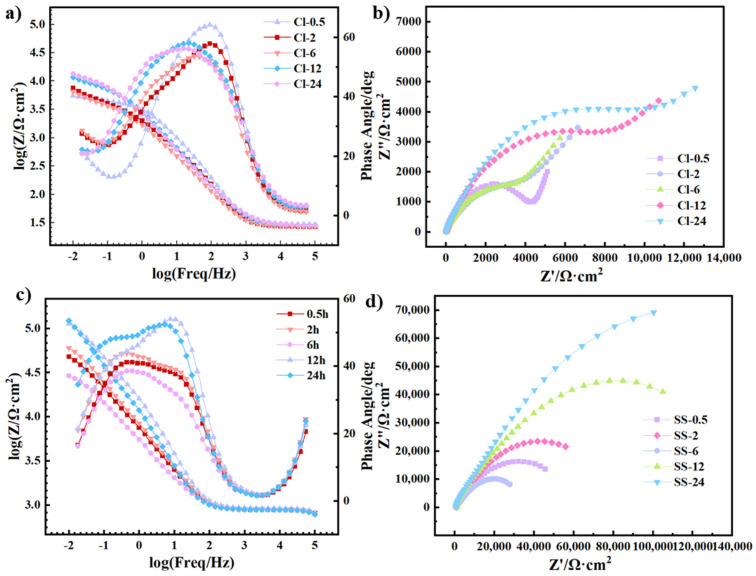
Nyquist plots and Bode plots for the EIS of different samples in (**a**,**b**) a NaCl solution or (**c**,**d**) a simulated archaeological soil solution. The solid curves represent the fitted results.

**Figure 10:**
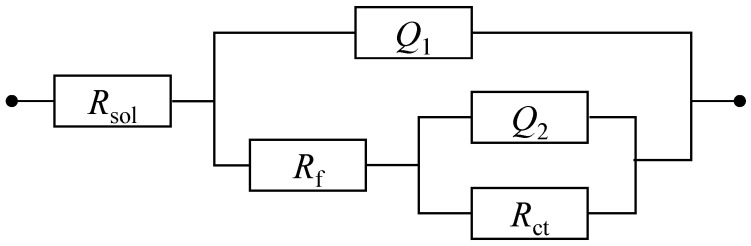
Equivalent circuits for electrochemical impedance spectroscopy data fitting.

Figure 9 shows the Nyquist and Bode plots of the Cu-Sn alloys in a 3.5% NaCl solution or a simulated archaeological soil solution. All EIS data were well fitted to the proposed equivalent circuit *R_s_* − (*Q*_1_*/*(*R_f_* − *Q*_2_*/R*_ct_)), as shown in Figure 10. The equivalent circuit model consists of electrolyte resistance (*R_sol_*), film resistance (*R_f_*), charge transfer resistance (*R*_ct_), and constant phase elements (*Q*_1_, *Q*_2_) that characterize the film capacitance and the double-layer capacitance. *Q*_2_ was used instead of the ideal two-layer capacitance (*C_dl_*) due to the non-ideal behavior of the non-uniform electrode [45,46,47]. In the NaCl solution, the Nyquist plot presented a small semicircle, and its diameter gradually increases over time, indicating a gradual improvement in the corrosion resistance of the alloy. In the simulated archeological soil solution, the diameter of the semicircle showed an overall increasing trend with time, and was larger, suggesting that the corrosion rust layers were more compact and hence had better corrosion resistance. The Bode plot in Figure 9b,d also support the findings of the Nyquist plot. In the NaCl solution, the |Z| value decreased significantly as the frequency decreased, indicating that the corrosion product layer provided limited protection; the phase angle curve exhibited a narrow peak, reflecting the relatively loose nature of the corrosion rust layers. Similarly, the |Z| value in the simulated archeological soil solution was higher, and the phase angle curve presented a broader peak range, which indicated that the corrosion product layer had better stability and protective properties.

The fitted results of the EIS data in Figure 9 are shown in Table 3. The *R*_f_ in the simulated archeological soil solution was generally higher than that in the NaCl solution, which means that the corrosion product layer in the simulated archeological soil solution was denser, effectively blocking the passage of ions. The corrosion rate was reduced. The *R*_ct_ in the simulated archeological soil solution was significantly higher and presented a more pronounced time dependence compared to the NaCl solution. The rapid charge transfer in NaCl environment means that the corrosion reactions were extremely active, and too many corrosion products fell off of the rough rust layer, resulting in uncontrolled cyclic corrosion. This is also the reason why the rust that forms in Cl^−^-containing environments has been regarded as harmful rust.

#### 3.3.4. Effect of Rust Layers Formed in Archaeological Soil Environments

**Figure 11:**
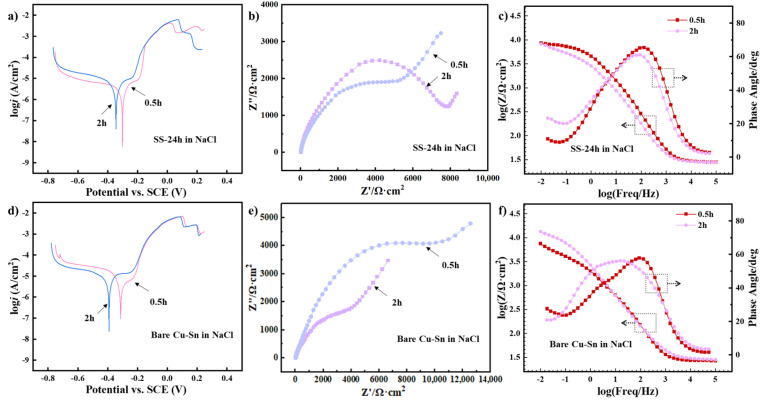
Tafel polarization and EIS curves of (**a**–**c**) SS-24 and (**d**–**f**) the Cu-Sn alloy in a 3.5% NaCl solution for 0.5 h and 2 h. The solid curves represent the fitted results.

In order to explore the corrosion behaviors of the rust layer in an archaeological soil environment, we further compared the corrosion behaviors of SS-24 and bare Cu-Sn alloys through Tafel and EIS; the results are presented in Figure 11 and Table 4 and Table 5. From the Tafel curves, it can be seen that the values of *i*_corr_ of SS-24 at 0.5 h and 2 h in the NaCl solution were 7.120 μA·cm^−2^ and 9.426 μA·cm^−2^, respectively, significantly lower than those of bare Cu-Sn alloy, which were 20.18 μA·cm^−2^ (0.5 h) and 25.58 μA·cm^−2^ (2 h). This suggests that the rust layer formed in the archaeological soil environment could protect the Cu-Sn alloy from external corrosive media. However, the *i*_corr_ of SS-24 increased slightly from 7.120 μA·cm^−2^ at 0.5 h to 9.426 μA·cm^−2^ at 2 h, and the *R*_p_ declined from 9.46 kΩ·cm^−2^ at 0.5 h to 6.85 kΩ·cm^−2^ at 2 h, indicating a gradual weakening of the passivation effect over time. That is to say, although SS-24 protected the Cu-Sn substrate, its protective effect gradually decreased as the rust layer progressively transformed into harmful rust (Cl^−^-containing corrosion products).

The Nyquist plot for SS-24 exhibited a larger impedance semicircle than that of the bare Cu-Sn alloys (Figure 11b,e), signifying that the rust layers possess stronger corrosion resistance than the bare Cu-Sn alloys, which corroborated the findings from the Tafel polarization parameters. As seen in the Bode plots (Figure 11c,f), there was difference in the |Z| values of SS-24 and the bare Cu-Sn alloys over time. As for SS-24, |Z| at 0.5 h increased significantly in the low-frequency region, suggesting a dense and protective corrosion product layer. However, the |Z| at 2 h decreased significantly, particularly in the low-frequency region, reflecting breakdown of the corrosion product layer and more severe corrosion. On the contrary, the |Z| of the bare Cu-Sn alloys increased over time, which contributed to corrosion products covering the substrate. The equivalent circuit fitting parameters (Table 5) showed that the *R*_ct_ values of SS-24 were greater than those of the Cu-Sn alloys, but the *R*_ct_ values decreased from 0.5 h to 2 h. Moreover, as the immersion time increased, the n_2_ values of the Cu-Sn alloys in the NaCl solution decreased from 0.528 to 0.426, and that of SS-24 in the NaCl solution increased from 0.531 to 0.825, indicating that the rust layers on the Cu-Sn alloys gradually reached a state of relative equilibrium. Specifically, the rust layer that formed in NaCl solution exhibited resistance (n_2_ = 0.528~0.426), while the rust layer that formed in the archaeological soil solution showed capacitance (n_2_ = 0.531~0.825), which was attributed to the latter being more compact and stable.

From the above analysis, it was concluded that the rust layer generated in the archeological soil environment had a protective effect on bronze, but that continuous Cl^−^ penetration reduced the corrosion resistance. The decrease in the protective effect of rust layers might be related to the surface changes of SS-24 when immersed in an NaCl solution. On one hand, Cl^−^ entered the rust layer and promote the generation of CuCl and Cu_2_(OH)Cl_3_, which resulted in a rough surface and induced damage by the corrosive medium to the substrate. On the other hand, the Cl^−^-containing corrosive products improved the wettability with an aqueous solution, facilitating deep diffusion of the corrosive medium into the rust layers, and thus the corrosive reactions became stronger over time.

### 3.4. The Corrosion Mechanism of Cu-Sn Alloy in Two Corrosive Environments

**Figure 12:**
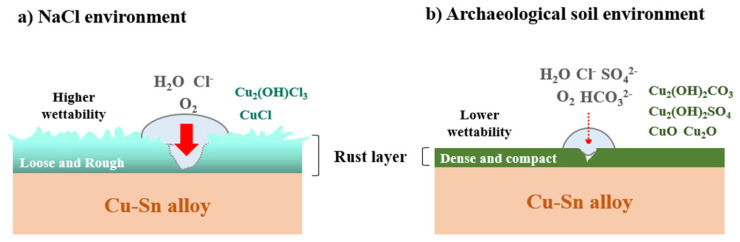
Schematic of the corrosion mechanism of Cu-Sn alloy after immersion in (**a**) 3.5% NaCl solution and (**b**) simulated archaeological-soil solution.

The previous sections compared the corrosion behavior of Cu-Sn alloys in two different corrosive media. Figure 12 illustrates the corrosion mechanisms of Cu-Sn alloys in a 3.5% NaCl solution and a simulated archaeological soil solution. Naturally, Cl^−^ ions play a dominant role in the corrosion process of Cu-Sn alloys in the 3.5% NaCl solution. Corrosion in this environment leads to the formation of CuCl and Cu_2_(OH)_3_Cl, and these products enhances the surface wettability of the alloy, facilitating penetration of water and oxygen, thereby accelerating the corrosion rate [48]. Additionally, the porous nature of Cu_2_(OH)_3_Cl and CuCl increases surface roughness, further reducing the contact angle and resulting in a positive feedback loop of hydrophilicity-induced accelerated corrosion [49,50]. In contrast, in the simulated archaeological soil solution, the corrosion behavior of the Cu-Sn alloys was influenced by multiple soil-derived ions, not only Cl^−^. These ions react with copper, forming a stable and dense corrosion product layer, consisting of CuO/Cu_2_O, Cu_2_(OH)_2_CO_3_, and Cu_2_(OH)_2_SO_4_ (Figure 12b) [24,51]. The rust layers formed in the simulated archaeological soil exhibited low porosity, and their dense structure not only hindered ion diffusion but also enhanced surface hydrophobicity by reducing the number of hydroxyl (-OH) groups [52,53].

Electrochemical analysis in the simulated archaeological soil solution demonstrated a positive shift in the potential (E, V) of the Cu-Sn alloy, indicating an enhancement of anodic polarization. This transformation may have resulted from the increased surface coverage of the dense rust layer, which restricted electrolyte penetration and metal ion dissolution [54]. Furthermore, the C_dl_ value of the hydrophilic surface (NaCl environment) was significantly higher than that of the hydrophobic surface (simulated archaeological soil environment), suggesting that the rough and porous rust layer facilitated electrolyte infiltration into its pores, thereby increasing the effective corrosion area of the substrate [55]. Compared with previous reports [5,8,10,24], this work discusses the interaction between corrosive media and bronze substrates from the perspective of wettability. In other words, in addition to removing powdery rust, the wettability modification of the bronze surface also plays an important role in inhibiting corrosion.

## 4. Conclusions

We prepared a simulated archaeological soil solution based on the soluble ion composition of archaeological soil around bronze ware unearthed in Shaanxi, and subsequently investigated and compared the corrosion behavior of the Cu-Sn alloy in NaCl and an archaeological environments.

(1) In a 3.5 wt.% NaCl solution, the *i*_corr_ of Cu-Sn alloys increased from 4.845 μA·cm^−2^ to 27.21 μA·cm^−2^, forming a loose and porous structure of CuCl and Cu_2_(OH)_3_Cl. In contrast, in the simulated archaeological soil solution, the *i*_corr_ remained below 1.6 μA·cm^−2^, and the rust layer was compact and stable, primarily composed of Cu_2_(OH)_2_CO_3_ and CuO.
(2) The stable rust layer exhibited lower roughness and a smaller contact angle than the unstable rust layer that formed in the NaCl environment, which significantly slowed further penetration of corrosive media and further enhanced corrosion resistance by about two orders of magnitude.
(3) EIS measurements and polarization curves showed that the *R*_ct_ in the simulated archaeological soil solution was significantly higher than that in the 3.5% NaCl solution (up to 416.7 kΩ·cm^2^), and the stability of OCP was significantly improved, indicating that the corrosion product layer possessed excellent passivation and protective properties.
(4) The alloy, when pre-corroded for 24 h in the simulated archaeological soil solution, exhibited a lower *i*_corr_ (0 h: 7.120 μA·cm^−2^, 2 h: 9.426 μA·cm^−2^) and a higher *R*_ct_ (0 h: 8.124 kΩ·cm^2^ h: 3.118 × 10^9^ kΩ·cm^2^) when subsequently immersed in an NaCl solution, indicating that the dense corrosion product layer formed during pre-corrosion can effectively delay the infiltration of Cl^−^.

## Figures and Tables

**Table 1:** The names of the samples immersed in the simulated archaeological soil solution or the NaCl solution for 0.5 h, 2 h, 6 h, 12 h, or 24 h.

Immersion Solution	Immersion Time (h)	Name
3.5 wt.% NaCl solution	0.5	Cl-0.5
	2	Cl-2
	6	Cl-6
	12	Cl-12
	24	Cl-24
Simulated archaeological soil solution	0.5	SS-0.5
	2	SS-2
	6	SS-6
	12	SS-12
	24	SS-24

**Table 2:** Fitted parameters for Figure 8. PPR: passivation potential range.

Sample	*E*_corr_ (V)	*i*_corr_ (μA·cm^−2^)	*R*_p_ (kΩ·cm^2^)	*β*_a_	*β*_c_	*i*_pass_ (A·cm^−2^)	PPR (V)
Cl-0.5	−0.250	4.845	5.17	14.13	3.24	0.00686	0.098~0.133
Cl-2	−0.278	25.58	4.00	0.74	3.51	0.00658	0.079~0.117
Cl-6	−0.292	15.28	4.12	2.77	4.14	0.00589	0.059~0.095
Cl-12	−0.289	23.32	3.91	0.75	4.02	0.00414	0.015~0.046
Cl-24	−0.282	27.21	3.27	1.02	3.87	0.00264	−0.027~0.011
SS-0.5	−0.073	1.441	25.31	2.97	8.95	-	-
SS-2	−0.074	1.183	27.56	3.27	10.07	-	-
SS-6	−0.076	0.796	35.83	5.40	9.85	-	-
SS-12	−0.066	0.807	39.05	4.56	9.23	-	-
SS-24	−0.060	1.588	24.40	4.20	7.02	5.2 × 10^−6^	−0.041~−0.016

**Table 3:** Fitted equivalent circuit parameters for Figure 9.

Sample	*R*_sol_ (Ω·cm^2^)	*Q*_1_ (μΩ^−1^s^n^/cm^2^)	n_1_	*R*_f_ (Ω·cm^2^)	*Q*_2_ (μΩ^−1^s^n^/cm^2^)	n_2_	*R*_ct_ (Ω·cm^2^)	χ2 (×10^−3^)
Cl-0.5	27.88	30.52	0.812	3.795 × 10^3^	1367	0.479	9.763 × 10^6^	0.64
Cl-2	22.23	235.9	0.323	2.894	21.16	0.826	2.452 × 10^4^	0.72
Cl-6	23.58	272.7	0.689	33.92	65.57	0.771	6.132 × 10^5^	0.92
Cl-12	24.73	302.5	0.672	4.068	74.15	0.814	7.534 × 10^4^	0.78
Cl-24	27.24	32.36	0.803	2.639	41.21	0.704	2422	0.94
SS-0.5	0.0035	6.528 × 10^−4^	0.965	836.1	46.60	0.595	6.288 × 10^5^	0.84
SS-2	0.0041	7.841 × 10^−4^	0.879	845.4	41.95	0.596	9.023 × 10^5^	0.73
SS-6	0.00044	7.471 × 10^−4^	0.986	839.2	67.87	0.575	3.992 × 10^6^	0.69
SS-12	912.7	7.363	0.869	15030	23.91	0.605	4.569 × 10^6^	0.91
SS-24	877.9	12.07	0.845	13930	29.34	0.613	2.735 × 10^4^	0.95

**Table 4:** Fitted polarization parameters for Figure 11a,c. PPR: passivation potential range.

Sample	Time (h)	*E*_corr_ (V)	*i*_corr_ (μA·cm^−2^)	*R*_p_ (kΩ·cm^2^)	*β*_a_	*β*_c_	*i*_pass_ (A·cm^−2^)	PPR (V)
SS-24	0.5	−0.252	7.120	9.46	4.41	2.04	0.00425	0.014–0.063
	2	−0.265	9.426	6.85	3.69	3.05	0.00602	0.074–0.230
Cu-Sn alloy	0.5	−0.212	20.18	2.23	1.92	3.37	0.00444	0.011–0.132
	2	−0.242	12.21	4.91	0.92	4.92	0.00658	0.083–0.117

**Table 5:** Fitted equivalent circuit parameters for Figure 11b,d.

Sample	Time (h)	*R*_sol_ (Ω·cm^2^)	*Q*_1_ (μΩ^−1^s^n^/cm^2^)	n_1_	*R*_f_ (Ω·cm^2^)	*Q*_2_ (μΩ^−1^s^n^/cm^2^)	n_2_	R_ct_ (Ω·cm^2^)	χ2 (×10^−3^)
SS-24	0.5	28.02	10.26	0.894	905.0	50.78	0.531	8124	0.93
	2	20.11	197.4	0.195	746.7	27.53	0.825	12118	0.85
Cu-Sn alloys	0.5	23.98	36.70	0.838	296.3	122.3	0.528	3884	0.87
	2	24.53	52.08	0.803	325.4	101.9	0.426	7971	0.96

## Data Availability

The data that support the findings of this study are available from the corresponding author upon request (due to privacy).

## Supplementary Materials

The following supporting information can be downloaded at: https://www.mdpi.com/article/10.3390/ma18061359/s1, Figure S1: Surface morphology of the uncorroded Cu-Sn alloy; Table S1: The element compositions of Cu-Sn alloys; Table S2: Soluble ion compositions of archaeological soil around bronze ware unearthed; Table S3: EDS results of Cu-Sn alloys treated in the NaCl solution and simulated archaeological-soil solution.
