# Supplementary material for: Comparative Study of the Corrosive Behaviors of Rust Layers on Bronze Ware in Different Corrosive Environments
Source: Materials (Basel). 2025 Mar 19;18(6):1359. doi: 10.3390/ma18061359 (PMC11943919; doi:10.3390/ma18061359)
Supplement: Supplementary file 1 [file materials-18-01359-s001.zip › materials-3505911-supplementary.pdf]

# Supporting information

# Comparative Study of the Corrosive Behaviors of Rust Layers on Bronze Ware in Different Corrosive Environments

**Bingbing Li [1,2], Qixing Xia [1,2,*] and Wenqiang Dong [1,2,*]**

[1] Cultural Heritage Research Institute, Northwestern Polytechnical University, Xi'an 710072, China; lllice2370@gmail.com
[2] Key Laboratory of Archaeological Exploration and Cultural Heritage Conservation Technology (Northwestern Polytechnical University), Ministry of Education, Xi'an 710129, China
* Correspondence: xqixing@nwpu.edu.cn (Q.X.); dongwq@nwpu.edu.cn (W.D.);
Tel.: +86-152-4467-0570 (Q.X.)

**Table S1.** The element compositions of Cu-Sn alloys

| **Element** | **Cu** | **Sn** | **Other** |
|---|---|---|---|
| Composition (wt.%) | ≥94 | 5~6 | ≤1 |

**Table S2.** Soluble ion compositions of archaeological soil around bronze ware unearthed

| **Soluble Ions** | **Results（mg/kg）** |
|---|---|
| $Na^+$ | 118.8 |
| $K^+$ | 22.5 |
| $Ca^{2+}$ | 148.7 |
| $Mg^{2+}$ | 18.5 |
| $SO_4^{2-}$ | 407.7 |
| $HCO_3^{2-}$ | 281.3 |
| $Cl^-$ | 37.4 |

**Table S3.** EDS results of Cu-Sn alloys treated in the NaCl solution and simulated archaeological-soil solution

| **Sample** | **Cu (at.%)** | **Cl (at.%)** | **O (at.%)** | **C (at.%)** | **Sn (at.%)** |
|---|---|---|---|---|---|
| Cl-2 | 71.4 | 14.5 | 12.1 | 1.9 | 0.1 |
| Cl-6 | 64.1 | 15.7 | 20.0 | 1.2 | 0.2 |
| Cl-12 | 62.2 | 22.1 | 14.9 | 0.7 | 0.1 |
| Cl-24 | 64.3 | 19.9 | 14.1 | 1.6 | 0.1 |
| SS-2 | 87.6 | 1.3 | 5.4 | 5.5 | 0.2 |
| SS-6 | 87.3 | 1.4 | 7.0 | 3.1 | 1.2 |
| SS-12 | 84.3 | 2.2 | 5.8 | 7.5 | 0.2 |
| SS-24 | 81.9 | 2.3 | 6.4 | 9.1 | 0.3 |

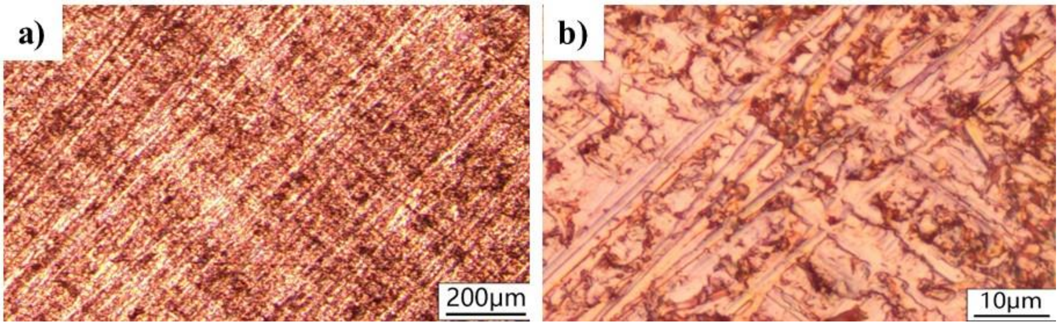


**Figure S1.** Surface morphology of the uncorroded Cu-Sn alloy
